# A Novel TRG-N Prognostic Classification System for Esophageal Cancer Undergoing Neoadjuvant Therapy Followed by Esophagectomy: A Study Based on the Netherlands Cancer Registry

**DOI:** 10.1097/SLA.0000000000006869

**Published:** 2025-09-23

**Authors:** Jingpu Wang, Zhouqiao Wu, Rob H.A. Verhoeven, Lucas Goense, Nadia Haj Mohammad, Stella Mook, Peter S.N. van Rossum, Marije Slingerland, Jan Erik Freund, Jelle P. Ruurda, Richard van Hillegersberg

**Affiliations:** * Department of Surgery, University Medical Center Utrecht, Utrecht, The Netherlands; † Key Laboratory of Carcinogenesis and Translational Research (Ministry of Education), Department of Gastrointestinal Surgery, Peking University Cancer Hospital and Institute, Beijing, China; ‡ Netherlands Comprehensive Cancer Organization (IKNL), Department of Research & Development, Utrecht, The Netherlands; § Amsterdam UMC location University of Amsterdam, Medical Oncology, Amsterdam, The Netherlands; ∥ Cancer Center Amsterdam, Cancer Treatment and Quality of Life, Amsterdam, The Netherlands; ¶ Department of Imaging and Cancer, Department of Medical Oncology, University Medical Center Utrecht, Utrecht University, Utrecht, The Netherlands; # Departments of Radiation Oncology, University Medical Center Utrecht, Utrecht University, Utrecht, The Netherlands; ** Department of Radiation Oncology, Amsterdam UMC, Amsterdam, The Netherlands; †† Department of Medical Oncology, Leiden University Medical Center, Leiden, The Netherlands

**Keywords:** esophageal cancer, prognostic classification system, tumor regression grade, ypTNM stage

## Abstract

**Objective:**

To develop a new prognostic classification system centered on tumor regression grade (TRG) and ypN stage that can effectively stratify overall survival (OS) of esophageal cancer patients undergoing neoadjuvant therapy followed by R0 esophagectomy.

**Background:**

Although the prognostic value of combining TRG and ypN stage has been demonstrated, a prognostic classification system integrating these factors, trained using large-scale data, remains unavailable.

**Methods:**

Data from the Netherlands Cancer Registry (2015–2022) were analyzed. A new TRG-N prognostic classification system for OS was developed by grouping patients based on cN stage, ypN stage, and TRG. The prognostic performance of the TRG-N classification was compared with the eighth edition AJCC ypTNM classification using 4 comparative metrics [log-rank χ², linear trend χ², Akaike information criterion (AIC), and C-index].

**Results:**

A total of 3193 patients were included. Among patients with adenocarcinoma, the TRG-N classification showed superior linear trend χ² and AIC to the ypTNM classification. However, the log-rank χ² of the TRG-N classification was inferior to that of the ypTNM classification, with no significant difference in the C-index (*P*=0.206) between the 2 systems. Among patients with squamous cell carcinoma, the TRG-N classification significantly outperformed the ypTNM classification in log-rank χ², linear trend χ², AIC, and C-index (*P*=0.018).

**Conclusions:**

The TRG-N classification demonstrated comparable prognostic performance to the AJCC ypTNM classification for esophageal adenocarcinoma but showed superior prognostic value for esophageal squamous cell carcinoma, making it a potentially more effective tool for risk stratification in esophageal cancer patients.

Esophagectomy is the mainstay of curative treatment for patients with resectable esophageal cancer.^1–4^ Multimodal treatment, which incorporates neoadjuvant therapy (NAT) alongside surgery, has become the standard of care for locally advanced esophageal cancer, as it offers a better prognosis compared with surgery alone.^5–7^ Accordingly, the eighth edition AJCC staging manual introduced the ypTNM prognostic classification system to stratify the survival of patients with esophageal adenocarcinoma or squamous cell carcinoma who undergo NAT followed by esophagectomy.^8,9^

Despite its prognostic utility, the ypTNM classification does not incorporate tumor regression grade (TRG), a histopathologic measure of tumor response to NAT. However, several studies have highlighted the prognostic value of combining TRG with ypN stage in both esophageal squamous cell carcinoma and adenocarcinoma, potentially outperforming the AJCC ypTNM classification.^10–13^

A study based on the Dutch population combined the Mandard TRG of the primary tumor with the ypN stage to create a prognostic classification system for esophageal adenocarcinoma.^12^ This resulted in the TRG-ypN score: TRG1-ypN0, TRG>1-ypN0, TRG1-ypN+, and TRG>1-ypN+. However, this classification was defined arbitrarily rather than being derived from real-world data through statistical analysis, which raised concerns about its rationale and validity.

Therefore, this study aims to develop a new prognostic classification system, using data from the Netherlands Cancer Registry (NCR), incorporating prognostically relevant clinical stages, ypN stage and TRG, and to improve risk stratification in esophageal cancer patients undergoing NAT followed by esophagectomy. The prognostic performance of this new system was compared with both the eighth edition AJCC ypTNM classification system and the Dutch TRG-ypN score in this study.^12^

## METHODS

This population-based study utilized prospective data from the NCR, a nationwide registry covering the 17.8 million residents of the Netherlands. Managed by the Netherlands Comprehensive Cancer Organization (IKNL), the NCR systematically records data on all newly diagnosed malignant tumors in the country.^14,15^ All data are recorded by trained data managers from medical records of all Dutch hospitals according to the NCR’s registration and coding manual. This study was approved by the Privacy Review Board of the NCR. Missing pathologic information for patients was supplemented using data from the PALGA database. Pathologic results in the Netherlands are digitally archived in PALGA, which has had nationwide coverage since 1991.^16^ The archive collects over 2 million new results annually from pathology laboratories, including cytology, histology, and autopsies.

### Patient Selection

All patients diagnosed with esophageal or gastroesophageal junction cancer between 2015 and 2022 who underwent NAT followed by surgery were extracted from the NCR. The exclusion criteria consisted of patients with (1) unknown histologic type; (2) non-esophagectomy surgical procedures; (3) fewer than 15 lymph nodes examined; (4) R1/R2 resection; (5) missing TRG/ypN stage information; (6) distant metastasis.

### Staging and Grading

Techniques used in the clinical staging of patients with non-metastatic esophageal cancer in the Netherlands include endoscopy with biopsy, integrated ^18^F fluorodeoxyglucose positron emission tomography (PET) with diagnostic-quality computed tomography (CT) scanning, and optionally endoscopic ultrasound, endoscopic bronchoscopic ultrasound or bronchoscopy.^15^ Patients registered in the NCR database were staged according to the seventh edition AJCC/UICC during 2015 to 2017 and staged from 2017 onwards according to the eighth edition. The Mandard TRG was used to assess the response of the primary tumor to NAT. The Mandard TRG is a 5-point scale: TRG1 indicates complete regression (fibrosis with no detectable tumor tissue); TRG2 represents fibrosis with scattered tumor cells; TRG3 denotes fibrosis with a predominance of fibrosis over tumor cells; TRG4 indicates fibrosis with a predominance of tumor cells; TRG5 signifies no regression, with the tumor tissue unchanged.^17,18^

### Treatment

In the Netherlands, neoadjuvant chemoradiotherapy according to the CROSS-regimen (23 fractions of 1.8 Gy to a total dose of 41.4 Gy, 5 times per week) using conformal external-beam radiotherapy combined with cycles of carboplatin administered weekly during 5 weeks (area under the curve 2 mg per ml per min) and paclitaxel (50 mg/m2) followed by esophagectomy is recommended as treatment for patients with resectable esophageal cancer (cT1b-4a N0-3 M0) since 2010.^19^ In addition, patients who received neoadjuvant chemotherapy, primarily those with esophagogastric junction adenocarcinoma, were also included in this study. The neoadjuvant chemotherapy regimens consisted of anthracycline triplets [epirubicin, capecitabin, and oxaliplatin (EOX); epirubicin, cisplatin, and 5-fluorouracil (ECF); epirubicin, capecitabin, and cisplatin (ECC); or epirubicin, oxaliplatin, and 5-fluorouracil (EOF)] and FLOT (fluorouracil, leucovorin, oxaliplatin, and docetaxel).^20^

### Outcome Measures

The primary outcome measure was overall survival (OS) from the date of surgery to the date of death or the final follow-up.

### Statistical Analysis

Clinical characteristics were described by using frequencies and percentages. Considering that the original stage of esophageal tumors before NAT may also be associated with patient survival, multivariable Cox regression analysis was used to identify independent prognostic variables from cT stage, cN stage, ypN stage, and TRG to construct an effective prognostic classification system.^21^ The Kaplan-Meier method and log-rank test were used to evaluate differences in OS across prognostic groups and to simplify the prognostic variables. All patients were categorized into different subgroups based on the identified independent prognostic variables. Multiple imputation was used to impute missing values and generate 20 new data sets. Multivariable Cox regression analysis was used to calculate the differences (ie, hazard ratios, HR) in all-cause mortality risk among the prognostic subgroups. The analysis was adjusted for sex, age, BMI, Charlson comorbidity score, PS score, ASA score, year of diagnosis, tumor location, histologic type, tumor differentiation, type of neoadjuvant therapy, type of resection, type of surgical technique, and the number of dissected lymph nodes. Subsequently, the subgroups with similar all-cause mortality risks were combined into one prognostic group, with the remaining groups forming the new TRG-N prognostic classification system. To compare the discriminatory ability (ie, differences in OS among patients with different stages), gradient monotonicity (ie, a trend where patients in earlier stages have longer survival than those in more advanced stages), and overall prognostic performance of the AJCC ypTNM classification, the Dutch TRG-ypN score, and the new prognostic classification, log-rank χ² test, linear trend χ² test, and AIC were computed, respectively. Higher log-rank χ² and linear trend χ² values indicate better discriminatory power and gradient monotonicity, respectively, whereas a lower AIC suggests a better model fit. The Concordance index (C-index) was calculated using 1000 bootstrap resamples to evaluate the discriminatory ability of the Cox models based on the 3 prognostic classification systems. The prognostic value of the 3 classification systems was also compared in subgroups based on histologic type, type of neoadjuvant therapy, type of resection, and number of dissected lymph nodes. A C-index closer to 1 indicates a higher predictive accuracy of the model. A 2-sided *P*<0.05 was considered statistically significant. All statistical analyses were performed by using SPSS version 25.0 software (SPSS, Chicago, IL) and R software version 4.3.3.

## RESULTS

### Patient Population

**FIGURE 1:**
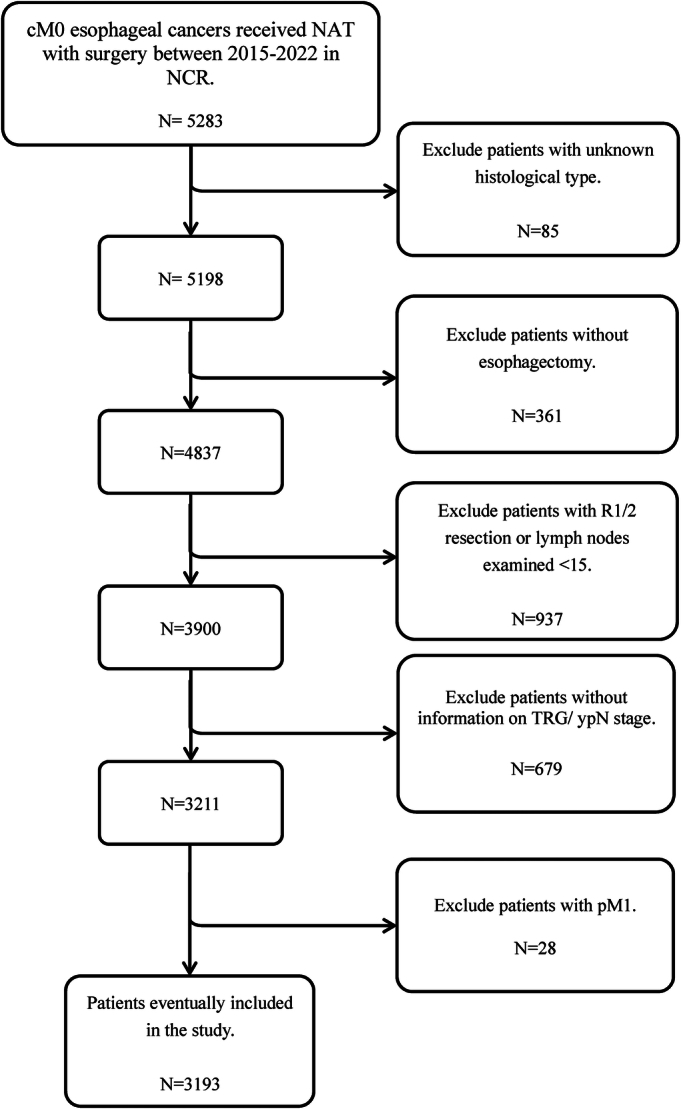
Selection process of patients.

A total of 5283 patients who underwent surgery between 2015 and 2022 were extracted, of whom 2090 patients were excluded, leaving 3193 included patients who underwent NAT followed by R0 esophagectomy (Fig. 1). The clinical characteristics of included patients are shown in Table 1. Of the 3193 included patients, 2572 (81%) had esophageal adenocarcinoma, whereas 621 (19%) had squamous cell carcinoma. A total of 204 patients (6%) received neoadjuvant chemotherapy, whereas 2989 (94%) underwent neoadjuvant chemoradiotherapy. The median number of lymph nodes examined was 25 (IQR 20–33). The median follow-up time for the included patients was 61.7 months (IQR 34.8–83.4).

**TABLE 1:** Clinical Characteristics of Included Patients Undergoing R0 Esophagectomy After Neoadjuvant Therapy

Variables	All (3193)
Age, n (%)	
18–64	1368 (42.8)
65–79	1773 (55.5)
≥80	52 (1.6)
Sex, n (%)	
Male	2500 (78.3)
Female	693 (21.7)
Charlson comorbidity score, n (%)	
0	1711 (53.6)
1	991 (31.0)
2+	415 (13.0)
Missing	76 (2.4)
PS score WHO, n (%)	
0	1772 (55.5)
1	1112 (34.8)
2+	86 (2.7)
Unknown	216 (6.8)
Missing	7 (0.2)
ASA score, n (%)	
I	229 (7.2)
II	1933 (60.5)
III	886 (27.7)
IV	28 (0.9)
Unknown	117 (3.7)
BMI, n (%)	
<25	1132 (35.5)
25–30	1122 (35.1)
≥30	478 (15.0)
Missing	461 (14.4)
Year of diagnosis, n (%)	
2015–2017	1373 (43.0)
2018–2020	1117 (35.0)
2021–2022	703 (22.0)
Histology, n (%)	
adenocarcinoma	2572 (80.6)
squamous cell carcinoma	621 (19.4)
Tumor location, n (%)	
Upper 1/3 esophagus	27 (0.8)
Middle 1/3 esophagus	359 (11.2)
Lower 1/3 esophagus	2546 (79.7)
EGJ	214 (6.7)
Unknown	47 (1.5)
Differentiation, n (%)	
Good differentiation	108 (3.4)
Moderate differentiation	1493 (46.8)
Poor differentiation	1071 (33.5)
Unknown	521 (16.3)
Type of neoadjuvant therapy, n (%)	
Chemotherapy	204 (6.4)
Chemoradiotherapy	2989 (93.6)
Type of resection, n (%)	
Transhiatal esophagectomy	312 (9.8)
Transthoracic esophagectomy	2881 (90.2)
Surgical technique, n (%)	
Open	305 (9.6)
Laparoscopic-assisted	2080 (65.1)
Robot-assisted	721 (22.6)
Unknown	30 (0.9)
Missing	57 (1.8)
No. dissected lymph nodes, n (%)	
15–22	1192 (37.3)
≥23	2001 (62.7)
cT stage, n (%)	
T1	28 (0.9)
T2	835 (26.2)
T3	2196 (68.8)
T4a	33 (1.0)
T4b	34 (1.1)
Tx	67 (2.1)
cN stage, n (%)	
N0	1305 (40.9)
N1	1175 (36.8)
N2	605 (18.9)
N3	82 (2.6)
Nx	26 (0.8)
ypT stage, n (%)	
T0	1014 (31.8)
Tis	7 (0.2)
T1	580 (18.2)
T2	564 (17.7)
T3	1006 (31.5)
T4a	18 (0.6)
Tx	4 (0.1)
ypN stage, n (%)	
N0	2058 (64.5)
N1	688 (21.5)
N2	308 (9.6)
N3	139 (4.4)
TRG (Mandard), n (%)	
TRG1	1019 (31.9)
TRG2	828 (25.9)
TRG3	665 (20.8)
TRG4	457 (14.3)
TRG5	224 (7.0)

EGJ indicates esophagogastric junction.

### Variable Screening and Simplification

Multivariable Cox regression analysis showed that, in addition to ypN stage and TRG, cN stage was also an independent prognostic factor for patients undergoing NAT followed by R0 esophagectomy (Supplementary Table 1, Supplemental Digital Content 1, http://links.lww.com/SLA/F558). Therefore, cN stage, ypN stage, and TRG were used to develop the new prognostic classification, which was named the TRG-N classification. In the survival analysis based on TRG, no significant difference (*P*=0.218) in OS was observed between TRG4 and TRG5, so they were merged in the TRG-N classification system (Supplementary Figure 1A, Supplemental Digital Content 1, Supplemental Digital Content 1, http://links.lww.com/SLA/F558). The survival analysis based on cN stage showed no significant difference (*P*=0.155) in OS between cN1 and cN2 (Supplementary Figure 1B, Supplemental Digital Content 1, Supplemental Digital Content 1, http://links.lww.com/SLA/F558). In addition, due to the limited number of cN3 patients (only 82), cN1-3 was simplified to cN+. The survival analysis based on ypN stage showed a significant difference in OS between all adjacent stages; therefore, ypN staging was not simplified (Supplementary Figure 1C, Supplemental Digital Content 1, Supplemental Digital Content 1, http://links.lww.com/SLA/F558).

### Construction of the TRG-N Prognostic Classification System

**FIGURE 2:**
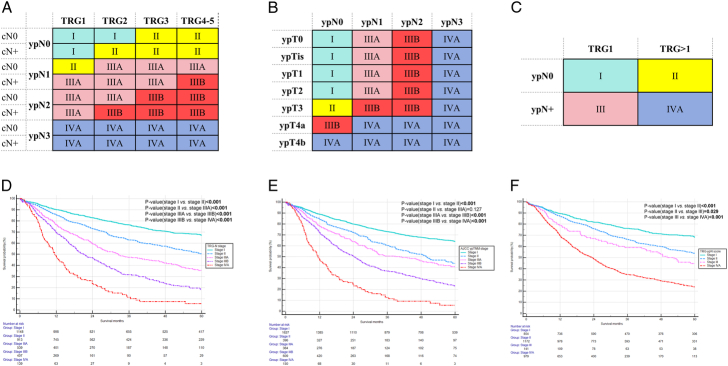
TRG-N prognostic classification system (A), eighth edition AJCC ypTNM prognostic classification system (B), and Dutch TRG-ypN score (C) for esophageal cancer patients undergoing esophagectomy after neoadjuvant therapy. Overall survival of all included esophageal cancer patients who underwent neoadjuvant therapy followed by R0 esophagectomy with different TRG-N stages (D), different AJCC ypTNM stages (E), and different TRG-ypN scores (F).

cN-ypN-TRG subgroups with similar all-cause mortality risk were merged into one prognostic stage: stage I [hazard ratio (HR): 1–1.2]; stage II (HR: 1.2–2.0); stage IIIA (HR: 2.0–3.0); stage IIIB (HR: 3.0–5.0); stage IVA (HR: >5.0) (Supplementary Table 2, Supplemental Digital Content 1, Supplemental Digital Content 1, http://links.lww.com/SLA/F558). The new TRG-N classification system is presented in Figure 2A, whereas the eighth edition AJCC ypTNM classification system for esophageal cancer and the Dutch TRG-ypN score are shown in Figures 2B, C, respectively.

### Comparison of Prognostic Classification Systems

All patients were staged according to the TRG-N classification, the AJCC ypTNM classification, and the Dutch TRG-ypN score, respectively, with their survival curves presented in Figure 2D–F. The new TRG-N classification demonstrated superior log-rank χ², linear trend χ², AIC, and C-index (*P*=0.012; *P*<0.001) compared with the ypTNM classification, and the TRG-ypN score (Table 2; Supplementary Figure 2A, Supplemental Digital Content 1, Supplemental Digital Content 1, http://links.lww.com/SLA/F558).

**TABLE 2:** Performance Indices of the Different Prognostic Classification Systems

Statistical Parameters	TRG-N Classification	Eighth AJCC ypTNM Classification	TRG-ypN Score
All patients (3146)			
Log-rank χ2	582.7*	568.9	414.7
Linear trend χ2	527.2*	494.8	395.5
AIC	22,077.0*	22,106.2	22,170.3
C-index (Bootstrap 95% CI)	0.650 (0.636–0.664)*	0.642 (0.628–0.656)	0.634 (0.620–0.648)
*P* (comparing C-index with TRG-N)	—	**0.012**	**<0.001**
Adenocarcinoma (2532)			
Log-rank χ2	469.0	478.7*	332.4
Linear trend χ2	426.2*	419.4	319.5
AIC	17,992.5*	17,999.8	18,070.0
C-index (Bootstrap 95% CI)	0.649 (0.634–0.665)	0.644 (0.629–0.660)	0.631 (0.615–0.646)
*P* (comparing C-index with TRG-N)	—	0.206	**<0.001**
Squamous cell carcinoma (614)			
Log-rank χ2	93.3*	58.8	59.7
Linear trend χ2	70.4*	50.5	51.4
AIC	2774.3*	2791	2788.7
C-index (Bootstrap 95% CI)	0.632 (0.597–0.668)*	0.611 (0.576–0.646)	0.621 (0.586–0.657)
*P* (comparing C-index with TRG-N)	—	**0.018**	**0.025**

Bold values are statistical significance of *P* < 0.05.

Better performance indices are shown by an asterisk (*).

In the subgroup analysis of patients with adenocarcinoma, the TRG-N classification showed superior linear trend χ² and AIC to the other 2 systems. However, its log-rank χ² was inferior to that of the ypTNM classification, and there was no significant difference in the C-index (*P*=0.206) between the TRG-N classification and the ypTNM classification (Table 2; Supplementary Figure 2B, Supplemental Digital Content 1, Supplemental Digital Content 1, http://links.lww.com/SLA/F558). The survival curves of the 3 classification systems are shown in Supplementary Figure 3, Supplemental Digital Content 1, Supplemental Digital Content 1, http://links.lww.com/SLA/F558.

In the subgroup analysis of patients with squamous cell carcinoma, the TRG-N classification outperformed the ypTNM classification, and the TRG-ypN score in log-rank χ², linear trend χ², AIC, and C-index (*P*=0.018; *P*=0.025) (Table 2; Supplementary Figure 2C, Supplemental Digital Content 1, Supplemental Digital Content 1, http://links.lww.com/SLA/F558). The survival curves of the 3 classification systems are shown in Supplementary Figure 4, Supplemental Digital Content 1, Supplemental Digital Content 1, http://links.lww.com/SLA/F558.

In the subgroups based on Lauren classification, type of neoadjuvant therapy, type of resection, and number of dissected lymph nodes, the TRG-N classification still demonstrated prognostic value comparable to, or even superior to, the ypTNM classification and the TRG-ypN score (Supplementary Table 3, Supplemental Digital Content 1, Supplemental Digital Content 1, http://links.lww.com/SLA/F558).

## DISCUSSION

This Dutch population-based study developed a novel TRG-N prognostic classification system based on cN stage, ypN stage, and TRG to stratify OS in patients with esophageal cancer undergoing NAT followed by R0 esophagectomy. The TRG-N prognostic classification system demonstrated superior prognostic value compared with the eighth edition AJCC ypTNM classification system for esophageal cancer and the previously published Dutch TRG-ypN score in this study.^12^ The subgroup analysis on patients with esophageal adenocarcinoma showed that the prognostic value of the TRG-N classification system was comparable to that of the ypTNM classification system. However, in patients with esophageal squamous cell carcinoma, the TRG-N classification system exhibited significantly better prognostic performance than ypTNM classification and the TRG-ypN score.

With multimodal therapy as the standard treatment for patients with resectable esophageal cancer, the eighth edition AJCC staging manual introduced the ypTNM classification system to stratify and predict survival in esophageal cancer patients undergoing NAT followed by esophagectomy.^8^ However, the accuracy and reliability of the ypT stage remain debatable, as it is defined by the maximum depth of tumor invasion and may fail to effectively predict outcomes after NAT due to the unpredictable distribution of tumor cells within the esophageal wall. This limitation may significantly compromise its prognostic value. TRG, as an assessment tool for the primary tumor after NAT, may be a more reliable option. Its prognostic value in both esophageal adenocarcinoma and squamous cell carcinoma has been demonstrated in multiple studies, which is consistent with the findings of this study.^10–12,22,23^ Even in 2 of them conducted on patients with esophageal squamous cell carcinoma, the prognostic value of the TRG-ypN combination was shown to surpass that of the eighth edition AJCC ypTNM prognostic classification system.^10,11^ The above evidence suggests that a prognostic classification system centered on TRG and ypN stage has the potential to replace the ypTNM prognostic classification system.

Interestingly, this study also found that incorporating cN stage could enhance the prognostic value of the TRG and ypN stage combination. A Chinese study based on patients with ypI stage esophageal squamous cell carcinoma who received trimodality therapy also demonstrated that adding cN stage could improve the prognostic performance of the eighth edition AJCC ypTNM classification.^21^ An international multicenter study on esophageal cancer patients undergoing trimodality therapy obtained similar results.^24^ This may be because the cN staging before NAT can complement the ypN staging after NAT, providing a better reflection on the risk of nodal and/or occult systemic metastasis, especially for patients whose lymph node metastases respond well to NAT. In this study, the TRG-N classification divided the cN stage into 2 categories: cN0 and cN+. However, the survival analysis based on cN stage indicated that cN3 should potentially be distinguished from cN1 to 2 and considered separately. Unfortunately, due to the limited number of patients with cN3, the patients were insufficient to train and improve the TRG-N prognostic classification system. Future studies may consider conducting a more in-depth investigation into this issue.

This study also indicated that histologic differences significantly affect the performance of the TRG-N classification system. This may be due to the varying sensitivity of esophageal squamous cell carcinoma and adenocarcinoma to radiotherapy and chemotherapy. In general, squamous cell carcinoma responds better to NAT (especially radiotherapy), with more significant tumor shrinkage and pathologic changes after treatment, which enhances the discriminatory ability and predictive power of the TRG-N classification in squamous cell carcinoma.^6,25^ In contrast, the treatment response in esophageal adenocarcinoma may be weaker or only partially responsive, resulting in poorer stratification with this classification in adenocarcinoma. Nevertheless, in this study, the TRG-N prognostic classification system still demonstrated prognostic value comparable to that of the AJCC ypTNM classification system in patients with adenocarcinoma.

Currently, various TRG systems are used worldwide for the pathologic evaluation of gastrointestinal tumors.^26^ These systems generally follow 2 key principles for grading tumor regression: first, the estimation of residual tumor in relation to fibrotic changes; and second, the estimation of residual tumor in relation to the previous tumor site, which can be expressed either as a percentage or in a descriptive manner.^27^ The Mandard TRG system is more commonly used in Western European countries; however, whether it holds the highest prognostic value and is the optimal choice for constructing the TRG-N classification system remains unclear.^17,26,27^ As TRG serves as the foundation of the TRG-N prognostic classification system, its definition may significantly impact the system’s performance. Further research is needed to identify the optimal TRG system, which is crucial for optimizing and promoting the application of the TRG-N prognostic classification system worldwide.

This study has the following limitations: first, the TRG-N prognostic classification system used in this study is based on the Mandard TRG, which limits its applicability in countries or regions that use other TRG systems; Second, the study was internally validated only in the Dutch cohort, and there is a lack of evidence supporting the applicability of the TRG-N classification in other populations; Third, 94% of the included patients received neoadjuvant chemoradiotherapy, which hinders the applicability of the TRG-N classification system in patients receiving neoadjuvant chemotherapy or immunotherapy; Fourth, the diagnostic modalities for cN staging were not uniformly defined, and their accuracy reflects the average level of clinical practice in the Netherlands; incorporating cN staging modalities may further optimize the TRG-N classification system in the future.

## CONCLUSIONS

A new prognostic classification system, the TRG-N classification, based on cN stage, ypN stage, and TRG, was developed to stratify OS in esophageal cancer patients undergoing NAT followed by R0 esophagectomy, trained on the Dutch population. In esophageal adenocarcinoma, the prognostic value of the TRG-N classification was comparable to that of the 8^th^ AJCC ypTNM classification, whereas in esophageal squamous cell carcinoma, it consistently demonstrated superior prognostic value.

## Supplementary Material

## DISCUSSANTS

### Paul M Schneider (Zurich, Switzerland)

Thank you, Dr. Wu, for your excellent presentation and for providing your revised manuscript. I would also like to thank the association for the privilege of discussing your paper. Thirty years after Mansard’s original publication and 20 years after the Cologne Group published a classification system based on tumor regression and ypN in *Ann Surg*, the topic is obviously still hot. Meanwhile, regression is included in the ypTNM Classification System, and indeed, tumor regression is undoubtedly important, as it is an objective parameter for treatment efficacy and has implications for postresection strategies for individual esophagogastric cancer patients. For example, the results of the SPACE FLOT trial, presented at ESMO in 2024, showed that if you have a complete response, you don’t need adjuvant treatment; you only need it for partial responders TRG 2 and 3. There is also considerable controversy around continuing the same treatment postoperatively when there is no clear evidence of an objective response to neoadjuvant therapy. In addition, there is another interesting study, the EORTC VESTIGE 1707 trial from 2024, which showed that the most important parameter in double adjuvant immunotherapy is the ypN category. As such, it is clear that we urgently need a robust and standardized classification system to address these important questions and to guide future research. Such a system would have significant implications for comparing various treatment modalities and tailoring individual treatment strategies. That said, several key questions remain unanswered, and I would like to raise 3 of them below.

First, the cN category is a very weak parameter in your “triple system”, with an accuracy of roughly 50% when endoscopic ultrasound and PET-CT is applied. Why did you include this parameter in your analysis?

Second, to the best of my knowledge, there have been no validation studies (eg, intraobserver and interobserver variation) for any regression system (eg, Mandard, Becker, Cologne, and others) in both neoadjuvant chemoradiation and chemotherapy for esophageal and/or gastric cancer. Should we continue with this, or start doing the groundwork?

Finally, the histopathologic response classification for the diffuse type, according to Laurén (WHO poorly cohesive type ± signet ring cells), remains unclear and is under investigation. Given this uncertainty, can this subgroup be reliably included in current clinical studies?

### Response From Zhouqiao Wu (Beijing, China)

Thank you very much for your questions. First, regarding the cN category, we all know that the clinical staging of lymph nodes is very difficult. As you said, it’s 50%. However, we included this in our analysis because, if we only select the YpN stage, then we are missing the previous information from the clinical staging. Of course, you can ask the radiologists and force them to provide very accurate clinical staging from N0 to N1 to N3. However, I would say that this is almost impossible because, to the best of our knowledge, it just isn’t accurate to provide this kind of diagnosis. So, what we did was to simply categorize them as negative or positive. Instead of providing very accurate 1, 2, and 3 clinical staging, we would simply state whether they were ±. Several studies have provided additional evidence showing that accuracy can improve modestly (around 70%). Our own statistical analysis supports this finding and indicates that it carries independent prognostic significance.

Your second question is also difficult. The regimens we use for the neoadjuvant therapy are quite different. We know that the Mandard classification system, which has been used in the Netherlands, has not intentionally been designed for all different kinds of neoadjuvant therapies. On the other hand, it is one of the few classification systems that we could use to evaluate this data. We conducted a subgroup analysis showing that this TRG system worked quite well, despite not being intended only for chemotherapy and so on. What we also know is that, in the international groups, there are some ongoing studies on a modified mandate TRG system. We hope it will perform better in the future. This can refer to a new question in the immunotherapy age. When we combine immunotherapy with these neoadjuvant regimens, it might provide other confounders, and it may require further modification of the TRG system we are using now. We will need to answer this through pathology and fundamental research. However, in the clinical practice that we are doing every day, we need a tool, and this is the best one we have.

Finally, with all this ongoing research, the current standard is the tool that we are using.

### Peter A Lodge (Leeds, United Kingdom)

This is an exciting study. How do you propose to validate what you’ve done?

### Response From Zhouqiao Wu (Beijing, China)

The first step for us would be to send this to Harvard and attempt to validate the data in a US cohort. However, the issue is that they don’t have the TRG registry in their national cohort. Now, we will look back and see whether there are any other European centers (we don’t necessarily need a national cohort) that can provide information to validate this. Of course, I’m also happy to bring this back to China and validate it in a large population. That’s the plan.
