# Supplementary material for: A Novel TRG-N Prognostic Classification System for Esophageal Cancer Undergoing Neoadjuvant Therapy Followed by Esophagectomy: A Study Based on the Netherlands Cancer Registry
Source: Ann Surg. 2025 Sep 23;282(5):837–44. doi: 10.1097/SLA.0000000000006869 (PMC12513032; doi:10.1097/SLA.0000000000006869)
Supplement: Supplementary file 1 [file sla-282-837-s001.docx]

**Table 1.** Multivariable Cox regression analysis for screening of independent prognostic variables for overall survival.

| **Variable** | **HR(95%CI)** | **p-value** |
| --- | --- | --- |
| cT stage |  |  |
| T1/2 | Reference | |
| T3 | 1.236(0.754,2.027) | 0.401 |
| T4a | 1.158(0.675,1.986) | 0.595 |
| T4b | 1.296(0.924,1.818) | 0.134 |
| cN stage |  |  |
| N0 | Reference | |
| N1 | 1.152(1.022,1.298) | **0.021** |
| N2 | 1.141(0.986,1.320) | 0.077 |
| N3 | 1.302(0.960,1.765) | 0.086 |
| TRG(Mandard) |  |  |
| 1 | Reference | |
| 2 | 1.397(1.204,1.620) | **<0.001** |
| 3 | 1.597(1.369,1.864) | **<0.001** |
| 4 | 1.655(1.394,1.964) | **<0.001** |
| 5 | 1.788(1.447,2.208) | **<0.001** |
| ypN stage |  |  |
| N0 | Reference | |
| N1 | 1.798(1.584,2.041) | **<0.001** |
| N2 | 2.577(2.194,3.027) | **<0.001** |
| N3 | 4.325(3.489,5.362) | **<0.001** |

HR, hazard ratio; TRG, tumor regression grade.

**Table 2.** All-cause mortality risk (i.e., hazard ratios, HR) among esophageal cancer patients undergoing R0 esophagectomy following neoadjuvant therapy across different cN-ypN-TRG subgroups.

|  |  | HR (Number of patients) | | | |
| --- | --- | --- | --- | --- | --- |
|  |  | **TRG1** | **TRG2** | **TRG3** | **TRG4-5** |
| cN0 | **ypN0** | 1(400) | 1.1(295) | 1.5(156) | 2.1(122) |
| cN+ |  | 1.1(468) | 1.9(279) | 1.9(191) | 1.9(131) |
| cN0 | **ypN1** | 1.6(36) | 3.1(66) | 2.9(57) | 3.0(71) |
| cN+ |  | 2.4(87) | 2.8(111) | 3.0(126) | 3.9(127) |
| cN0 | **ypN2** | NA (3) | 3.0(9) | 4.8(21) | 5.4(42) |
| cN+ |  | 2.7(13) | 3.9(44) | 4.7(70) | 5.0(103) |
| cN0 | **ypN3** | NA (0) | NA (4) | 6.1(9) | 11.6(14) |
| cN+ |  | NA (5) | 7.7(15) | 11.8(24) | 8.5(65) |

The adjusted variables included sex, age, BMI, Charlson comorbidity score, PS score, ASA score, year of diagnosis, tumor location, histological type, tumor differentiation, type of neoadjuvant therapy, type of resection, type of surgical technique, and the number of dissected lymph nodes

**Table 3.** Performance indices of the different prognostic classification systems in different subgroups.

| **Statistical parameters** | **TRG-N classification** | **8th AJCC ypTNM classification** | **TRG-ypN score** |
| --- | --- | --- | --- |
| **Intestinal adenocarcinoma** **(1654)** | | | |
| Log rank χ2 | 325.5 | 333.9 | 222.3 |
| Linear trend χ2 | 303.0 | 296.8 | 217.3 |
| AIC | 10639.1 | 10645.1 | 10703.0 |
| C index (Bootstrap 95% CI) | 0.662(0.643-0.681) | 0.657(0.638-0.676) | 0.637(0.619-0.656) |
| P-value (comparing C index with TRG-N) | - | 0.318 | **<0.001** |
| **Non-intestinal adenocarcinoma (463)** | | | |
| Log rank χ2 | 105.2 | 105.4 | 84.3 |
| Linear trend χ2 | 90.9 | 91.4 | 75.6 |
| AIC | 2689.9 | 2689.2 | 2699.1 |
| C index (Bootstrap 95% CI) | 0.650(0.616-0.684) | 0.647(0.612-0.682) | 0.636(0.602-0.670) |
| P-value (comparing C index with TRG-N) | - | 0.717 | 0.066 |
| **Neoadjuvant chemotherapy (198)** | | | |
| Log rank χ2 | 56.1 | 60.2 | 33.2 |
| Linear trend χ2 | 44.7 | 48.8 | 32.0 |
| AIC | 777.8 | 772.5 | 787.7 |
| C index (Bootstrap 95% CI) | 0.708(0.653-0.762) | 0.711(0.659-0.762) | 0.669(0.618-0.720) |
| P-value (comparing C index with TRG-N) | - | 0.786 | **0.001** |
| **Neoadjuvant chemoradiotherapy** **(2948)** | | | |
| Log rank χ2 | 558.5 | 532.1 | 390.0 |
| Linear trend χ2 | 499.5 | 459.5 | 370.6 |
| AIC | 20633.4 | 20670.1 | 20725.9 |
| C index (Bootstrap 95% CI) | 0.649(0.635-0.664) | 0.640(0.625-0.654) | 0.633(0.618-0.648) |
| P-value (comparing C index with TRG-N) | - | **0.004** | **<0.001** |
| **Number of dissected lymph nodes <23 (1180)** | | | |
| Log rank χ2 | 255.6 | 246.6 | 193.3 |
| Linear trend χ2 | 236.7 | 228.8 | 186.7 |
| AIC | 7719.8 | 7729.3 | 7754.6 |
| C index (Bootstrap 95% CI) | 0.660(0.638-0.682) | 0.652(0.631-0.674) | 0.645(0.623-0.667) |
| P-value (comparing C index with TRG-N) | - | 0.156 | **<0.001** |
| **Number of dissected lymph nodes ≧23 (1966)** | | | |
| Log rank χ2 | 343.2 | 339.4 | 229.8 |
| Linear trend χ2 | 297.8 | 273 | 212.9 |
| AIC | 12341.4 | 12362.7 | 12403.7 |
| C index (Bootstrap 95% CI) | 0.645(0.627-0.664) | 0.636(0.618-0.655) | 0.627(0.609-0.646) |
| P-value (comparing C index with TRG-N) | - | **0.041** | **<0.001** |
| **Transhiatal esophagectomy (305)** | | | |
| Log rank χ2 | 69.0 | 77.7 | 48.4 |
| Linear trend χ2 | 61.3 | 65.9 | 46.4 |
| AIC | 1571.1 | 1567 | 1581.4 |
| C index (Bootstrap 95% CI) | 0.668(0.625-0.711) | 0.674(0.631-0.717) | 0.649(0.607-0.691) |
| P-value (comparing C index with TRG-N) | - | 0.607 | **0.023** |
| **Transthoracic esophagectomy (2841)** | | | |
| Log rank χ2 | 514.3 | 493.3 | 371.3 |
| Linear trend χ2 | 466.0 | 429.3 | 349.7 |
| AIC | 1571.1 | 1567.0 | 1581.4 |
| C index (Bootstrap 95% CI) | 0.648(0.633-0.663) | 0.638(0.623-0.653) | 0.632(0.617-0.647) |
| P-value (comparing C index with TRG-N) | - | **0.005** | **<0.001** |


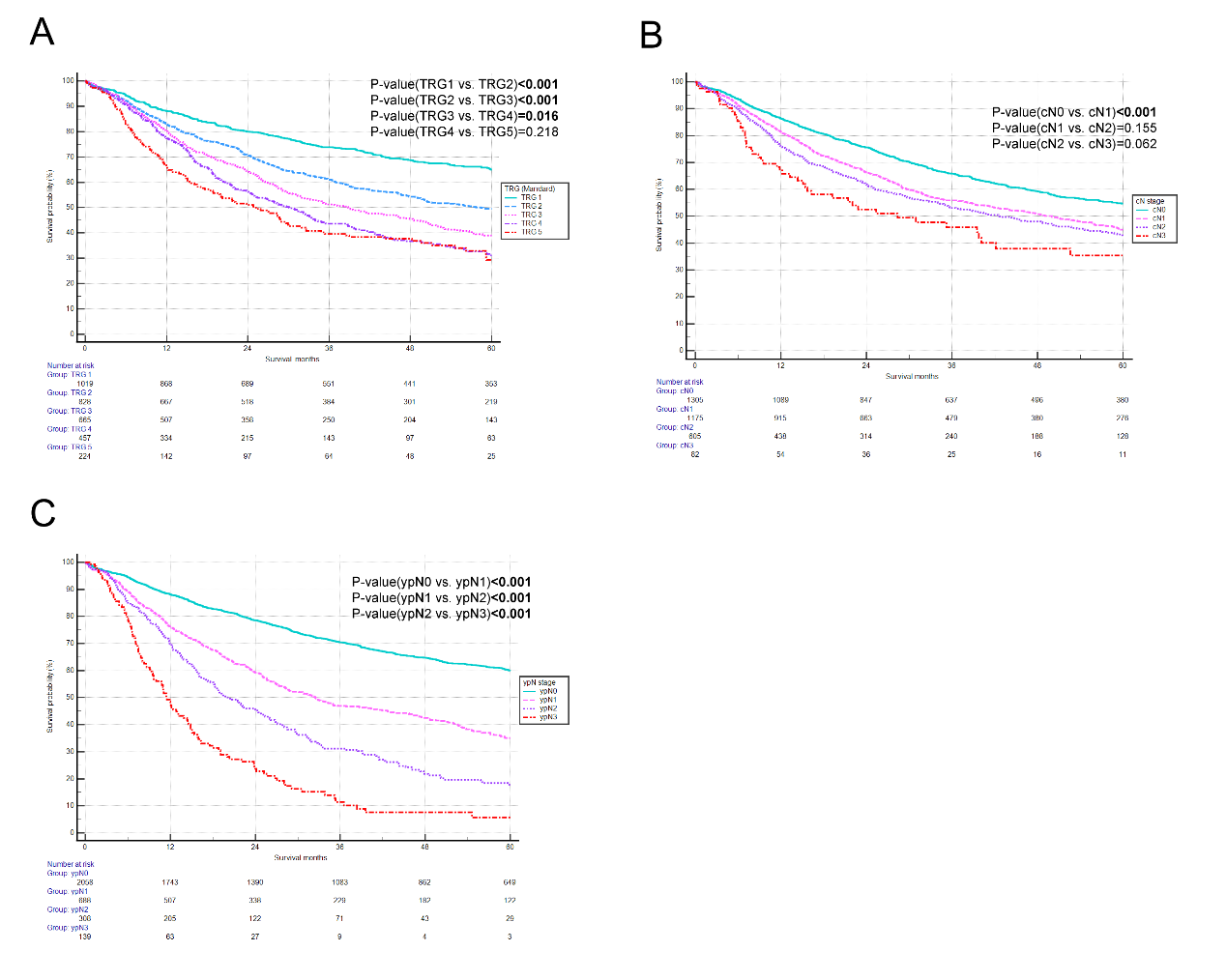


**Figure 1.** Overall survival of the esophageal cancer patients who underwent R0 esophagectomy following neoadjuvant therapy with different TRG (A), cN stages (B), and ypN stages (C). TRG, tumor regression grade.


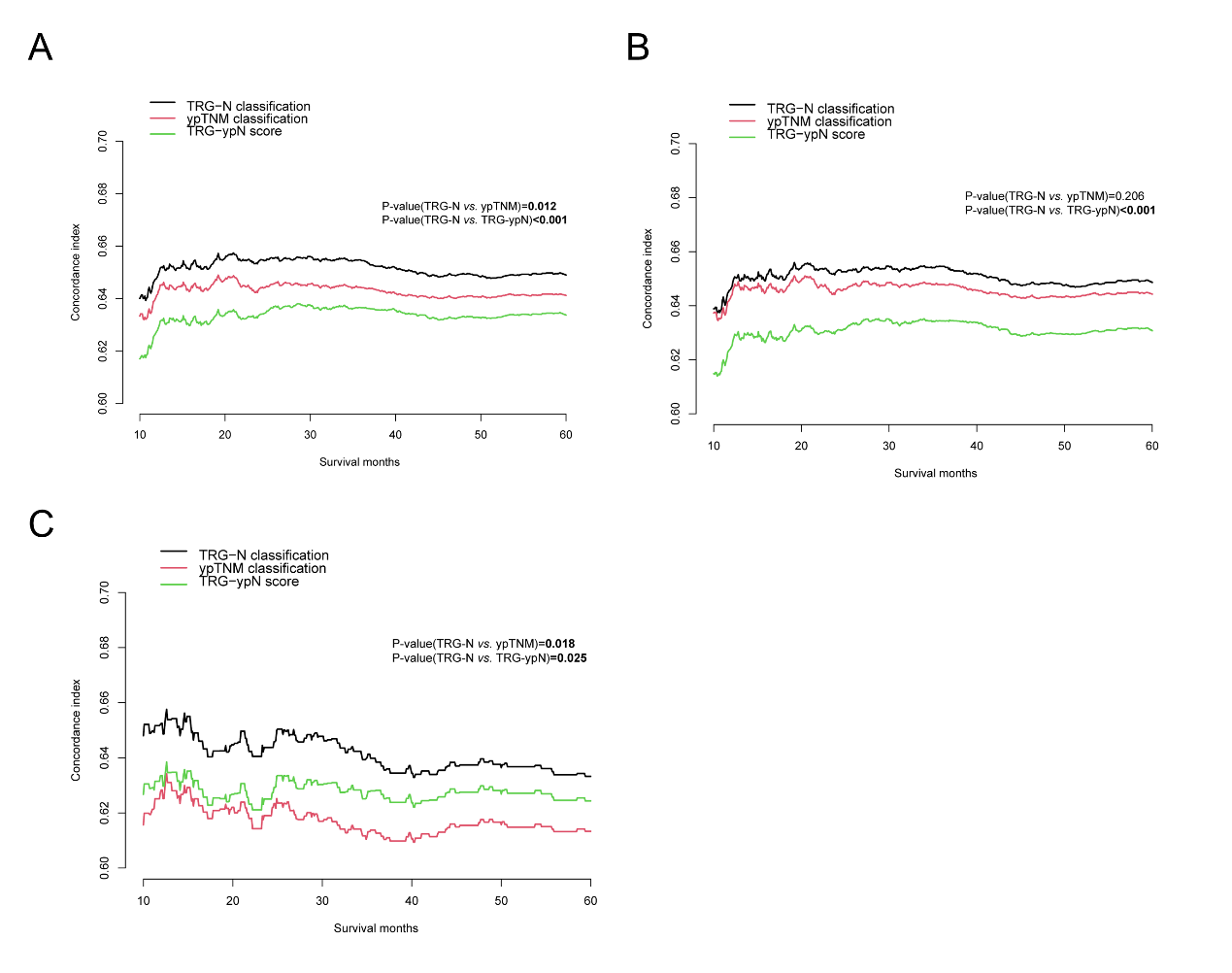


**Figure 2.** Time-dependent C-index of the TRG-N prognostic classification system, the 8th edition AJCC ypTNM prognostic classification system, and the Dutch TRG-ypN score in all esophageal cancer patients (A), esophageal adenocarcinoma patients (B), and esophageal squamous cell carcinoma patients (C) who underwent neoadjuvant therapy followed by esophagectomy. TRG, tumor regression grade.


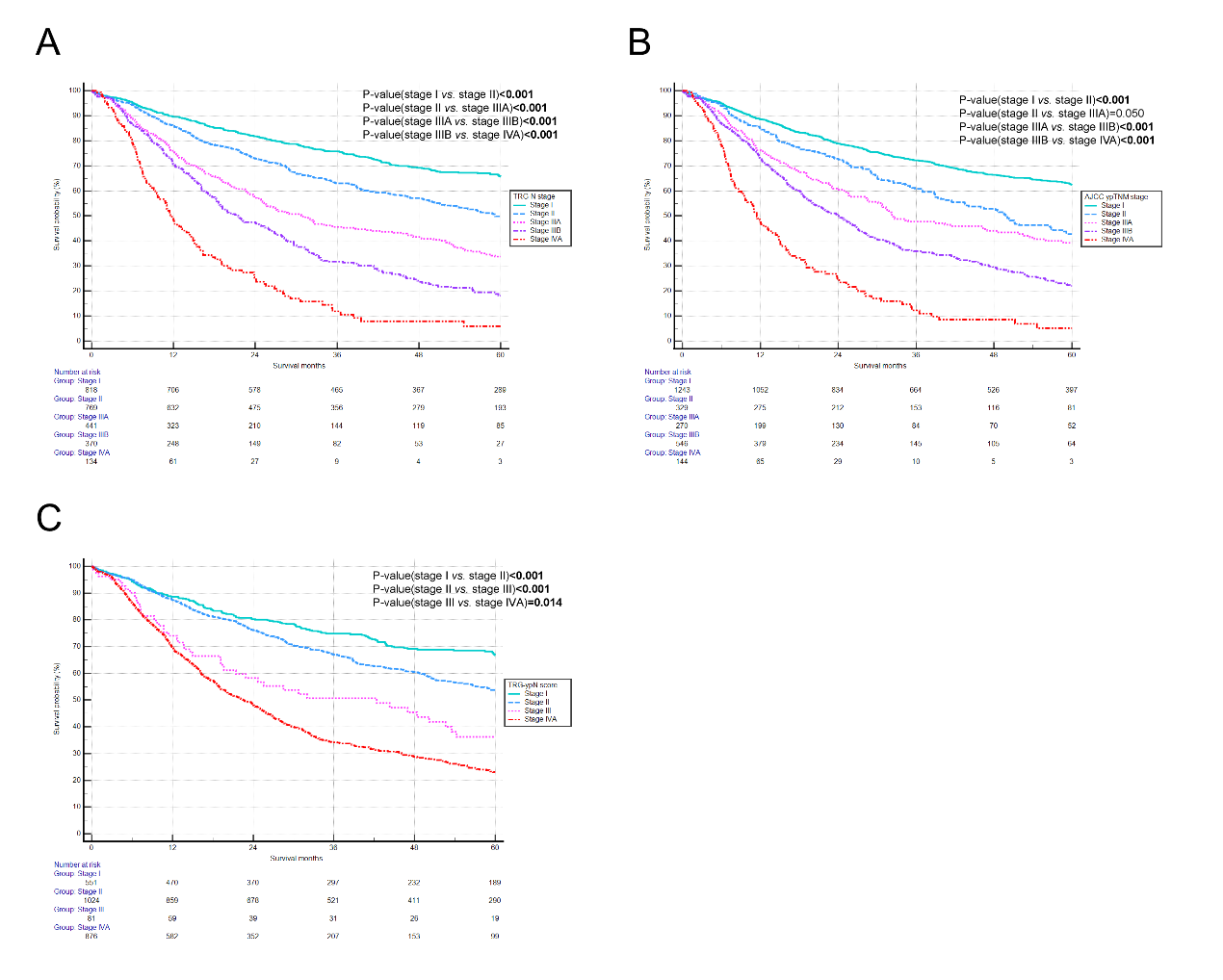
 **Figure 3.** Overall survival of the esophageal adenocarcinoma patients who underwent neoadjuvant therapy followed by esophagectomy with different TRG-N stages (A), different AJCC ypTNM stages (B), and different TRG-ypN scores (C). TRG, tumor regression grade.


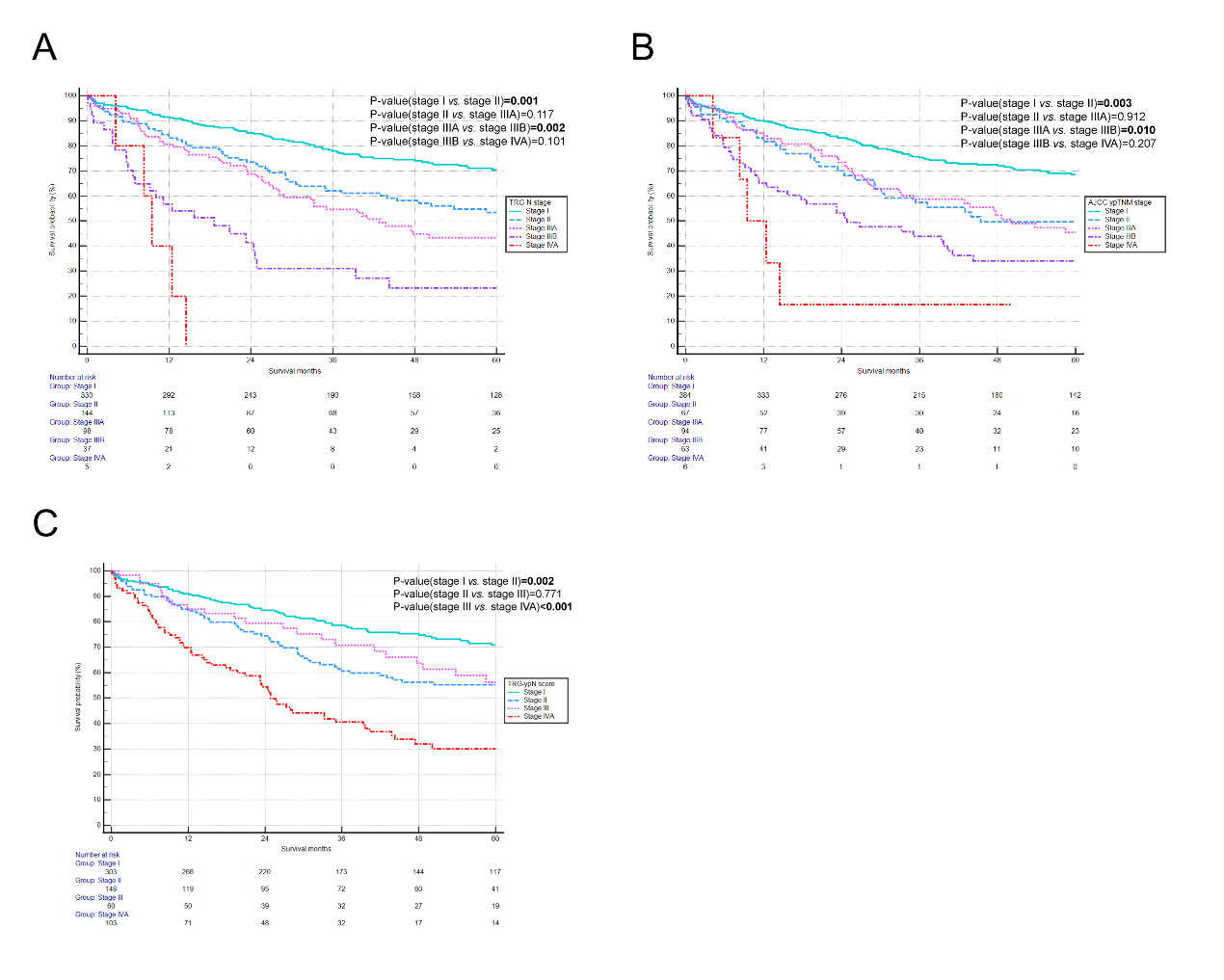
 **Figure 4.** Overall survival of the esophageal squamous cell carcinoma carcinoma patients who underwent neoadjuvant therapy followed by esophagectomy with different TRG-N stages (A), different AJCC ypTNM stages (B), and different TRG-ypN scores (C). TRG, tumor regression grade.
